# Proteomics analysis of human mesenchymal stromal/stem cell sarcomagenesis model identifies ALDH1A3 and CD99 as potential targets in the transformation process

**DOI:** 10.1186/s12915-025-02498-z

**Published:** 2026-01-09

**Authors:** Jonathan M. Gobin, Jun Gao, Veronica Rey, Juan Tornín, Gauri Muradia, Hala Halabi, Clara Bueno, Mercedes Guerrero-Murillo, Belen Lopez-Millan, Pablo Menendez, Michael Rosu-Myles, Rene Rodriguez, Jessie R. Lavoie

**Affiliations:** 1 Department of Biochemistry, Microbiology and Immunology, Faculty of Medicine, University of Ottawa, 451 Smyth Road, Ottawa, ON K1H 8M5 Canada; 2 Centre for Oncology, Radiopharmaceuticals and Research, Biologic and Radiopharmaceutical Drugs Directorate, Health Products and Food Branch, Health Canada, Ottawa, ON K1A 0K9 Canada; 3 Instituto de Investigacion Sanitaria del Principado de Asturias (ISPA) - Hospital Universitario Central de Asturias, Oviedo, 33011 Spain; 4 Instituto Universitario de Oncologia del Principado de Asturias, Oviedo, 33006 Spain; 5 Centro de Investigación Biomédica en Red-Oncología (CIBERONC), Instituto de Salud Carlos III, Madrid, 28029 Spain; 6 Josep Carreras Leukaemia Research Institute, Department of Biomedicine, School of Medicine, University of Barcelona, Barcelona, Spain; 7 Terapias Avanzadas (TERAV), Instituto de Salud Carlos III (ISCII) (RICORS, RD21/00170029) Madrid, Spain; 8 Red Española de Terapias Avanzadas (TERAV) - Instituto de Salud Carlos III (ISCII) (RICORS, RD21/0017/0029), Barcelona, Spain

**Keywords:** MSC-sarcoma model, Sarcomagenesis, Proteomics, CD99, ALDH1A3

## Abstract

**Background:**

Mesenchymal stromal/stem cells (MSC) may represent the cell-of-origin for sarcoma development. A collection of human MSCs sequentially mutated with an increasing number of oncogenic hits served to recreate a step-wise process of sarcomagenesis. To identify potential protein targets of interest in the MSC-sarcoma transformation process, quantitative mass spectrometry-based (LC–MS/MS) proteomics was performed.

**Results:**

Among the protein hits identified as significantly regulated in the transformation process, ALDH1A3 and CD99 were selected and further studied. Both ALDH1A3 abundance levels and activity were significantly upregulated in early-phase (immortalized) and fully transformed (sarcoma forming) cells as compared to normal MSCs. Inversely, CD99 total protein and cell-surface abundance levels were downregulated in immortalized and transformed MSCs. Downregulated CD99 was also identified in several human bone and soft tissue sarcoma subtypes.

**Conclusions:**

Proteomics investigation of a MSC-transformation model of sarcoma has yielded ALDH1A3 and CD99 as potential targets for sarcomagenesis that may contribute to a greater understanding of the disease and the development of novel therapeutic approaches.

**Supplementary Information:**

The online version contains supplementary material available at 10.1186/s12915-025-02498-z.

## Background

Sarcomas represent an extensive group of malignant diseases that affect mesodermal tissues such as bones, muscles, cartilage, or fat. It has been demonstrated that the stem cell population responsible for the homeostasis of these tissues, the mesenchymal stem/stromal cells (MSCs), may be the target cell that receives the first oncogenic insults and initiates sarcoma transformation [1]. Therefore, in previous work, we harnessed an available collection of sequentially mutated bone marrow-derived human MSCs (BM-hMSCs) [2] to generate different cell-of-origin sarcoma models [3, 4]. In this step-wise transformation model, wild-type BM-hMSCs were sequentially transformed by targeting them with up to five oncogenic mutations: hTERT (human Telomerase Reverse Transcriptase) overexpression (MSC-H1), p53 (MSC-H2) and Rb (retinoblastoma) inactivation (MSC-3H), c-myc (cellular myelocytomatosis oncogene) stabilization (MSC-H4), and introduction of H-RAS^v−12^ (Harvey Rat sarcoma virus) (MSC-5H) [2]. In addition, the fusion oncogene FUS-CHOP (CCAAT/enhancer-binding protein homologous protein) (FC), characteristic of myxoid liposarcoma (MLS), or the corresponding GFP-control, was ectopically expressed in all the MSC types (Additional file 1: Table S1) [3, 5]. MSC-3H-GFP and MSC-4H-GFP cells were immortalized in vitro but were unable to form sarcomas when inoculated in vivo, while MSC-4H-FC, MSC-5H-GFP, and MSC-5H-FC were fully transformed, demonstrating both in vitro immortalization and a capacity to form sarcomas in immunodeficient mice. The type of sarcoma arising in vivo was determined by the type of oncogenic insults introduced with FC driving the formation of tumours with the histological and molecular features of human MLS [3, 5].

This cell-of-origin model has demonstrated its utility to improve our understanding of the mechanisms governing sarcomagenesis. The model has been used to characterize the evolution of subpopulations of cancer cells that have acquired stem cell-like properties and are responsible for tumor dissemination and drug resistance [6–8]. Among the factors identified as potential markers of these cancer stem cell (CSC) subpopulations in sarcomas, several members of the aldehyde dehydrogenase (ALDH) family of detoxifying enzymes, such as ALDH1A1 and ALDH1A3, have been related to the malignant potential of CSCs [6, 8] and chemoresistance [9]. Moreover, MSC-based models of sarcoma have been useful for designing and testing specific therapies able to target the tumor populations that initiate, sustain, and expand tumor growth [10–15].

Here, we took advantage of this MSC-sarcoma transformation model and performed quantitative mass spectrometry-based proteomics comparing relative protein expression levels in normal, immortalized, and fully transformed MSCs to identify early markers of sarcomagenesis. Proteomics identified significantly upregulated and downregulated proteins between the mutated cell groups, among which ALDH1A3 and CD99 showed significant increased and decreased abundance levels, respectively. Verification of ALDH1A3 activity and CD99 cell surface abundance levels corroborated these results. Furthermore, staining of biopsies from various sarcoma subtypes confirmed that the decreased CD99 abundance levels observed in our BM-hMSC-sarcoma model was also indicative of expression patterns in native sarcomas. Overall, this work provides potential markers of sarcomagenesis that could broaden our understanding of the disease and which may contribute to the development of novel therapeutic approaches.

## Results

### Mass spectrometry-based proteomics analysis highlights key proteins related to MSC transformation process

**Fig. 1:**
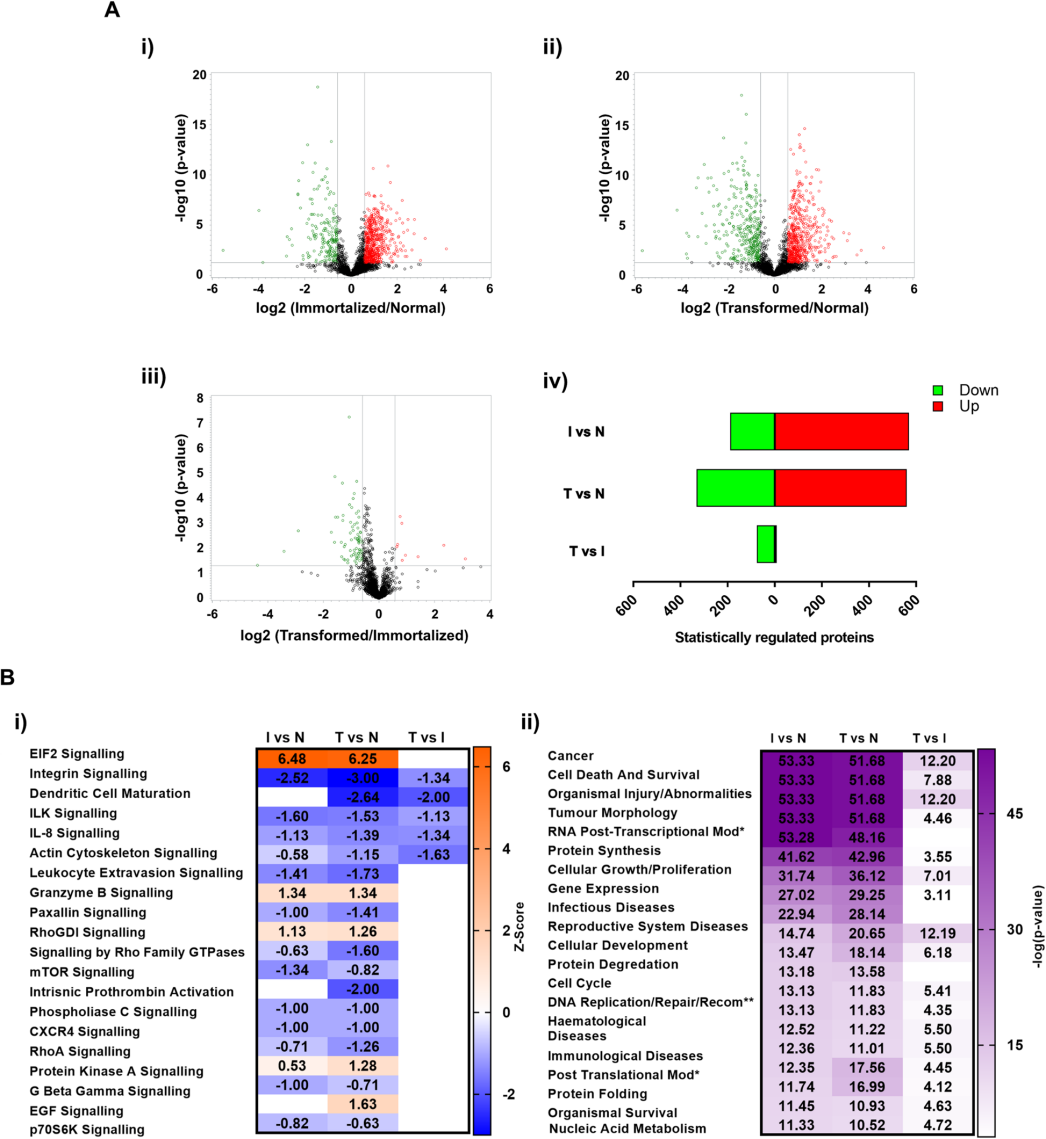
Mass spectrometry-based proteomics identifies statistically significant differentially regulated proteins at the different transformation stages of a MSC-based sarcoma model.** A** Volcano plots of each mass spectrometry dataset identifying the distribution of significantly differentially regulated proteins (log2 fold change ± 1.50, log *p*-value > 1.30 or raw *p*-value of < 0.05) between immortalized (i) or transformed MSCs (ii) compared to normal MSCs, and transformed compared to immortalized MSCs (iii). Statistically differentially regulated proteins (log2 fold change ± 1.50, log *p*-value > 1.30 or *p*-value of < 0.05) from each dataset (I vs N, T vs N and T vs I) (iv) comparing the upregulated and downregulated protein profiles between the groups. **B** (i) Top 20 most affected canonical pathways predicted by Ingenuity Pathway Analysis (IPA). Activation (orange) and repression (blue) of canonical pathways is based on the directional abundance ratio of proteins identified from the mass spectrometry datasets. (ii) Top 20 most significantly related diseases and functions predicted by IPA based on the directional abundance ratio of proteins identified from the mass spectrometry datasets. Log (*p*-value) is represented by increasing intensity of purple showing increased significance

In order to identify proteins with significantly altered abundance levels related to tumorigenic transformation, quantitative mass spectrometry-based proteomics using Tandem Mass Tags (TMT) labelling was performed on MSCs previously subjected to stepwise mutations [2, 3] (Fig. 1). In this study, protein lysates were isolated from wild type (normal, N) BM-hMSCs (bone marrow-derived human MSCs) derived from 3 healthy donors (hBM12A = N1; hBM15A = N2; hBM#44 = N3) and one set of cultures that were transduced with GFP to control for the potential effects of this vector on protein expression (hBM#44-GFP = N4). The protein abundance level in these cells was compared with lysates derived from three immortalized (I) BM-hMSC lines (MSC-3H-GFP = I1; MSC-3H-FC = I2; MSC-4H-GFP = I3) and three fully transformed (T) BM-hMSC lines (MSC-4H-FC = T1; MSC-5H-GFP = T2; MSC-5H-FC = T3) (Additional File 1: Table S1). Three separate proteomics datasets were generated where immortalized vs. normal (I vs N) (Additional File 2: Table S2), transformed vs. normal (T vs N) (Additional File 3: Table S3), and transformed vs. immortalized (T vs I) (Additional File 4: Table S4) were compared to identify significantly altered proteins using fold changes of ± 1.50 between conditions and a raw *p*-value of less than 0.05.

Volcano plots were generated from each comparison (I vs N; T vs N; T vs I) to compare the relative abundance of all proteins within each cell type. These data are demonstrated in Fig. 1 where the distribution of statistically altered proteins for each comparison is shown in the upper right (+ 1.50 fold change) and upper left corners (− 1.50 fold change) (Fig. 1A i-iii). Statistical analysis of the I vs N dataset showed a greater number of significantly upregulated proteins (570 proteins) as compared to the number of downregulated proteins (189 proteins) among a total of 2585 commonly identified proteins (Fig. 1A iv). Statistical analysis of the T vs N dataset showed a consistent distribution of significantly upregulated (561 proteins) and downregulated proteins (331 proteins) among a total of 2584 commonly identified proteins between datasets (Fig. 1A iv). Finally, statistical analysis of the T vs I dataset showed a low number of altered proteins where only 9 proteins were significantly upregulated as compared to 75 downregulated proteins among a total of 2584 commonly identified proteins (Fig. 1A iv).

As a validation of this proteomic analysis, we first used the mass spectrometry dataset to identify proteins corresponding to the oncogenic factors that were introduced to create the genetically modified MSC lines (Additional File 2: Table S2). As expected, telomerase reverse transcriptase (TERT) was identified as upregulated in both the immortalized (fold change of 17.37, *p* = 0.0023) and the transformed (fold change of 26.40, *p* = 0.0018) groups as compared to normal MSCs. Similarly, the RNA-binding protein FUS partner in the fusion oncoprotein FUS-CHOP was also identified as upregulated in immortalized (fold change of 3.36, *p* = 0.000082) and transformed (fold change of 3.63, *p* = 0.000046) groups as compared to normal MSCs. Finally, HRas proto-oncogene (HRas) was identified as upregulated in transformed MSCs as compared to normal (fold change of 3.28, *p* = value 0.0118) and immortalized MSCs (fold change of 2.65, *p* = value 0.0218).

Ingenuity Pathway Analysis (IPA) of proteins differentially expressed also revealed a great overlap between the signaling pathways (Fig. 1B i) and biological processes (Fig. 1B ii) altered in immortalized and transformed MSCs in comparison with normal MSCs. Altered pathways include the activation of EIF2 signaling, probably related to the inactivation of Rb in immortalized and transformed MSCs, and the inhibition of integrin, ILK, or IL-8-mediated signaling among other signaling pathways. Most affected biological processes are related to cancer, cell death, and survival or tumor morphology. Among the few pathways specifically altered in transformed vs normal but not immortalized vs normal cells, IPA analysis revealed the upregulation of EGF signaling, most likely related to the expression of mutant RAS in transformed MSCs, and the inhibition of dendritic cell maturation process and prothrombin activation (Fig. 1B i).

**Fig. 2:**
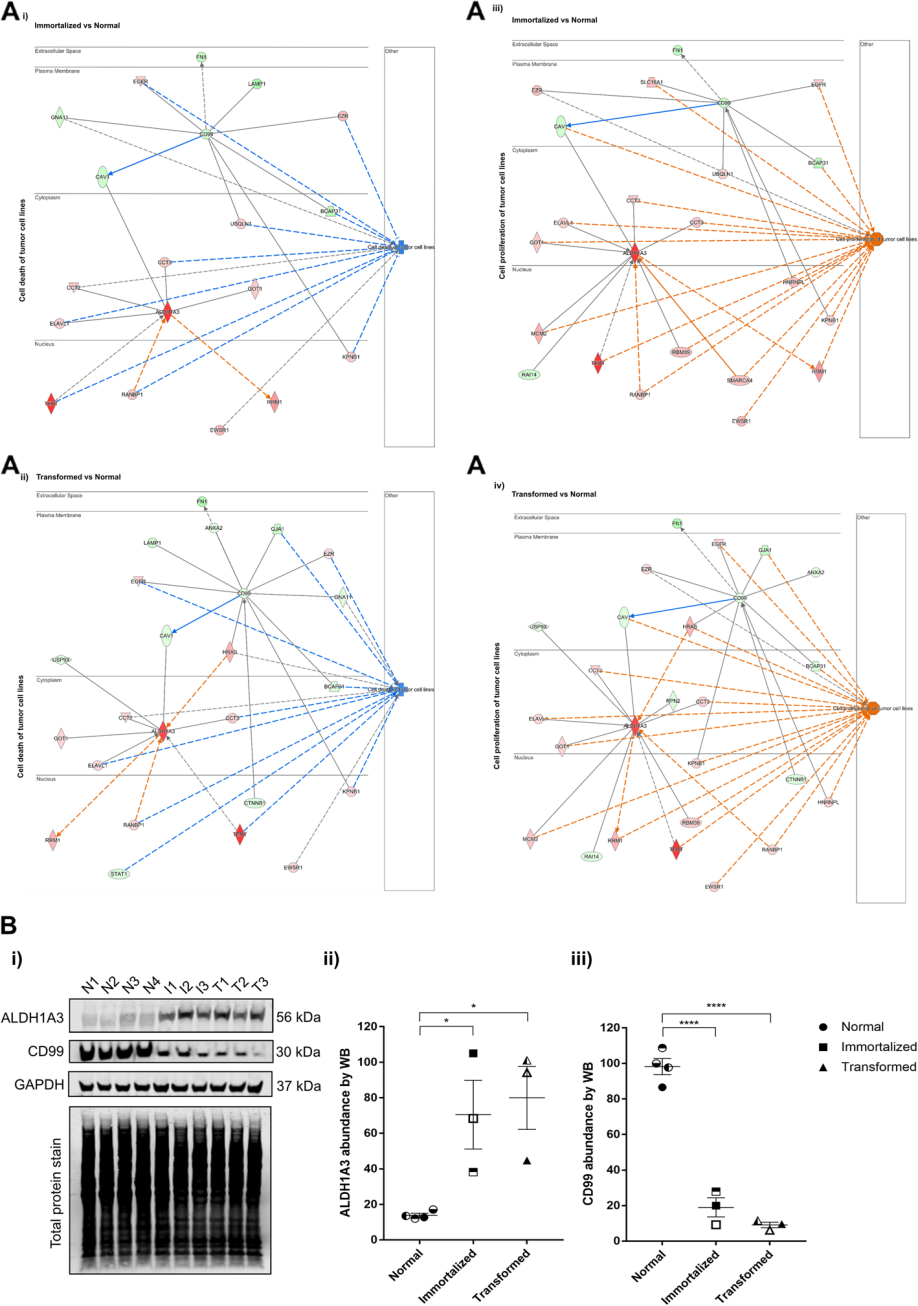
ALDH1A3 and CD99 participate in sarcomagenesis-related processes. A) Ingenuity Pathway Analysis (IPA) analysis shows ALDH1A3 and CD99 molecular interactions with key proteins involved in the overall inhibition of cell death of tumor cell lines (i-ii) and overall activation of cell proliferation of tumor cell lines (iii-iv). Predicted activation or inhibition states were inferred based on the expression values in the dataset, using IPA’s curated molecular interaction database and upstream regulator analysis. **B** Representative Western blot images showing ALDH1A3 and CD99 abundance levels in normal (*n* = 4; N1–N4), immortalized (*n* = 3; I1–I3), and transformed (*n* = 3; T1–T3) BM-hMSC samples. GAPDH and REVERT total protein stain is shown at the bottom and served as normalization controls (i). Quantification of ALDH1A3 abundance ratio comparing normal (*n* = 4; N1–N4), immortalized (*n* = 3; I1–I3), and transformed (*n* = 3; T1–T3) BM-hMSC samples (ii). (*N* = 3 independent experiments, * = *p*-value < 0.05). Full-length blots are presented in Figure S1. Quantification of CD99 abundance ratio comparing normal (*n* = 4; N1–N4), immortalized (*n* = 3; I1–I3), and transformed (*n* = 3; T1–T3) MSC samples (iii). (*N* = 3 independent experiments, **** = *p*-value < 0.0001). Statistical significance determined by one-way ANOVA post hoc Tukey’s test

Among the significantly upregulated proteins (fold change of > 1.50) from the proteomics datasets (Additional files 3–5: Tables S3-5), aldehyde dehydrogenase 1 family member A3 (ALDH1A3) was found to be upregulated in both the immortalized (fold change of 6.69 and FDR adj. *p*-value of 6.41 × 10^−5^) and the transformed (fold change of 8.04 and FDR adj. *p*-value of 3.90 × 10^−4^) groups as compared to the normal MSCs. In contrast, Cluster of Differentiation 99 (CD99) was among the downregulated proteomics dataset (fold change of < − 1.50) in the immortalized (fold change of − 1.63 and FDR adj. *p*-value of 0.0321) and the transformed (fold change of − 1.94 and FDR adj. *p*-value of 0.0080) groups as compared to the normal MSCs. Since ALDH1A3 is a pro-stemness factor in sarcomas [6, 8, 9] and CD99 is a hallmark of Ewing’s sarcoma [16] and a tumor suppressor in osteosarcoma [17], we selected these two candidate proteins for further validation as potential targets associated with the sarcoma transformation process. In both dataset comparisons (I vs N and T vs N), ALDH1A3 and CD99 were indirectly involved in the suppression of tumor cell death (Fig. 2A i and ii) and the activation of cell proliferation of tumor cell lines (Fig. 2A iii and iv). CD99 downregulation was associated with several intermediates, including epidermal growth factor receptor (EGFR), ezrin (EZR), karyopherin subunit beta-1 (KPNB1), B-cell receptor-associated protein 31 (BCAP31), and ubiquilin-1 (UBQLN1), which contributed to the predicted inhibition of tumor cell death (Fig. 2A i and ii). ALDH1A3 was linked to downstream targets such as T-complex protein 1 subunit gamma (CCT3), ELAVL1, Ran-specific binding protein 1 (RANBP1), and TERT, all of which are implicated in reducing apoptotic processes in tumor cells (Fig. 2A iii and iv). In the transformed datasets (Fig. 2A ii and iv), HRAS was identified as an upstream regulator contributing to the upregulation of ALDH1A3, potentially reinforcing both the increased proliferation and reduced cell death phenotypes characteristic of malignant transformation.

In both datasets (I vs N and T vs N), ALDH1A3 and CD99 were key contributors to the activation of cell proliferation of tumor cells (Fig. 2A iii and iv). ALDH1A3 was upregulated and connected to a network of effectors including Ribonucleotide Reductase M1 (RRM1), Minichromosome Maintenance protein 2 (MCM2), TERT, RANBP1, RNA-binding motif protein 39 (RBM39), SWI/SNF Related BAF Chromatin Remodeling Complex Subunit ATPase 4 (SMARCA4), Glutamic-oxaloacetic transaminase (GOT1), ELAVL1, T-complex protein 1 subunit beta (CCT2), and CCT3, which collectively supported cell proliferation. In the transformed group, HRAS also emerged as a contributor, with predicted (dashed orange) activation links to both ALDH1A3 and the cell proliferation function, suggesting it may modulate this pathway indirectly. Meanwhile, CD99 was downregulated in both datasets (Fig. 2A iii and iv). Its downregulation was indirectly associated with the upregulation of EGFR and the downregulation of GJA1, both known to enhance tumor cell proliferation. GJA1 was connected to cell proliferation via a dashed orange line ending in a T-bar, signifying a predicted activation of proliferation due to the loss of an inhibitory effect. This suggests that GJA1 normally acts to suppress proliferation, and its downregulation, potentially linked to the loss of CD99, removes this suppression, thereby contributing to tumor cell growth.

Next, Western blot analysis of proteins CD99 and ALDH1A3 was conducted on whole cell lysates of the MSC cell lines to validate the protein abundance ratios observed by mass spectrometry from the MSC transformation model (Fig. 2B i) (Additional file 6: Figure S1). Relative abundance ratios of ALDH1A3 showed significantly upregulated levels from immortalized (fold change of 5.10, *p* = 0.0433) and transformed (fold change of 5.71, *p* = 0.0220) MSC lines as compared to normal MSCs (Fig. 2B ii). Inversely, CD99 showed significant downregulated abundance levels from immortalized (fold change of − 5.26, *p* < 0.0001) and transformed (fold change of − 10.75, *p* < 0.0001) MSC lines as compared to normal MSCs (Fig. 2B iii). These Western blot results validated the observed alterations in CD99 and ALDH1A3 abundance levels detected by mass spectrometry and provided confidence to proceed with further validation of their activity linked to the MSC transformation process.

### Increase of ALDH1 activity and decrease of CD99 cell surface presence during the MSC transformation process

**Fig. 3:**
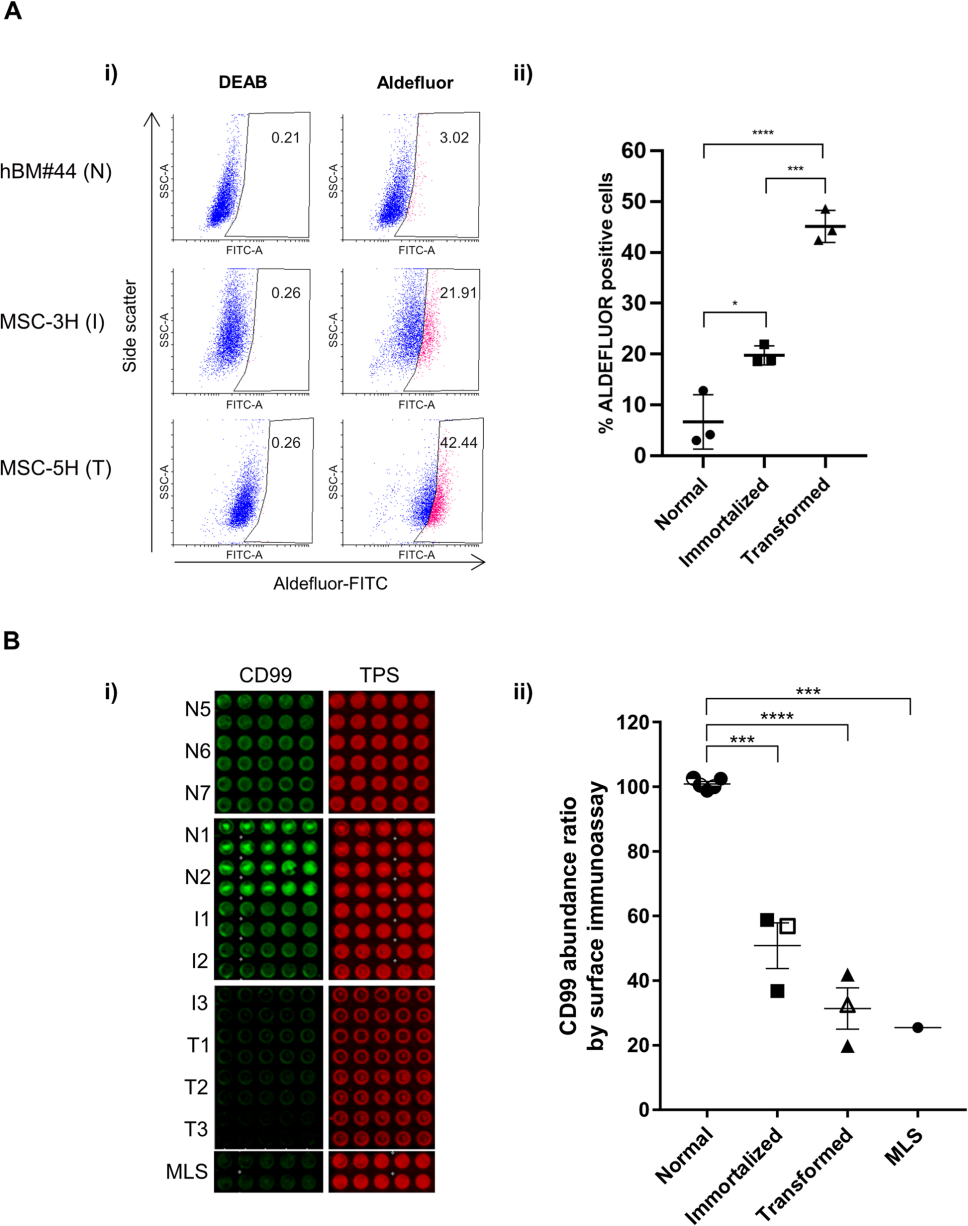
Confirmation of a concomitant increase in ALDH1 activity and decreased CD99 cell surface levels as MSCs progress towards fully transformed sarcoma-forming cells.** A** ALDEFLUOR assay showing the activity of ALDH1 in BM-MSC-44 (N3), MSC-3H (I3 prior to the transduction with a GFP-expressing vector), and MSC-5H (T2 prior to the transduction with a GFP-expressing vector) cells. ALDH1 activity was blocked with the specific inhibitor DEAB to establish the basal level. A representative assay (i) and the summary of 3 independent experiments (ii) are presented. Statistical significance determined by one-way ANOVA post hoc Tukey’s test. (* = *p*-value < 0.05; ** = *p*-value < 0.01). **B** Analysis of cell surface immunoassay detection of CD99 of normal (*n* = 5; N1–2 and N5–7), immortalized (*n* = 3; I1–I3), and transformed (*n* = 3; T1–T3) BM-hMSC samples, and mixoid liposarcoma (MLS) patient-derived cell line (*n* = 1). (i) A representative image of CD99 cell surface immunostaining using an Infrared Imaging System Odyssey CLx from Li-Cor. (ii) Graphical representation of CD99 abundance displayed as % value normalized to highest fluorescent intensity (*N* = 3 independent experiments each having 10 technical replicates, **** = *p*-value < 0.0001). Statistical significance determined by one-way ANOVA followed by Tukey’s test

Using the ALDEFLUOR assay, which uses a freely diffusing fluorescence ALDH1 substrate to measure the enzymatic activity of this family of detoxifying proteins, we previously showed that ALDH1A3 is the most prevalent isoform contributing to ALDH1 enzymatic activity. To confirm that the increased expression of ALDH1A3 in immortalized and transformed MSCs correlated with increased enzymatic activity, we performed an ALDEFLUOR assay with different MSC cultures. Because this assay is based on the generation of a green fluorescent compound, we compared the ALDEFLUOR activity of a wild-type MSC culture (N3) with that of the original version of MSC-3H (similar to I1) and MSC-5H cells (similar to T2) which have not been transduced with GFP-expressing lentiviral vectors. ALDH1 activity levels were extremely low in normal MSCs with only 3.86 ± 1.19% of cells staining positive with the ALDEFLUOR assay (Fig. 3A i-ii). By comparison, ALDH1 activity was significantly upregulated upon immortalization with 20.25 ± 2.35% of cells staining positive. Increased ALDH1 activity correlated even further with complete transformation as cells with the capacity to form tumours in mice showed a twofold increase in ALDEFLUOR positive cells (45.51 ± 4.34%). Thus, analysis of ALDH1 activity in our sarcoma model validates the upregulation of ALDH1A3 expression in immortalized and transformed MSCs and suggests increased ALDH1 activity could provide a promising indicator of MSC transformation towards a sarcoma phenotype.

As CD99 is a cell surface protein, we next validated abundance levels of CD99 on intact MSCs using whole cell immunostaining. CD99 abundance levels on normal MSCs and immortalized and transformed cell lines were compared with that of patient-derived myxoid liposarcoma (MLS) sample as a further form of validation (Fig. 3B) (Additional file 7: Fig S2). No significant difference in CD99 staining was identified between immortalized and transformed cell lines and patient MLS samples further supporting the relevance of our sarcoma model. Conversely, both the immortalized and transformed cell lines showed a significant reduction in surface CD99 abundance levels (*p* < 0.0001) compared to normal MSCs. These results validated the decreased abundance level of CD99 detected in immortalized and sarcoma-forming cells analyzed by mass spectrometry and Western blot and further showed that the decreased abundance of total CD99 protein is further reflected at the surface of these cells. Overall, the validation of altered abundance of ALDH1A3 and CD99 using ALDH function assays and cell surface immunostaining enforces the potential relevance of these proteins to the MSC transformation process and as potential sarcomagenesis markers.

The expression of both CD99 and ALDH1A3 was also interrogated in passage 0 primary MSC cultures established from pediatric (*n* = 3) and adult (*n* = 4) healthy BM aspirates [18, 19] using single-cell transcriptomics **(**Additional file 8: Figure S3). Single cell RNA sequencing (scRNAseq) analysis revealed expression of ALDH1A3 in < 10% of healthy BM-derived MSC while practically all these cells express CD99 (Additional file 8: Figure S3A-B), largely validating the proteomic results.

### Liposarcoma tissues present low abundance levels of CD99

**Fig. 4:**
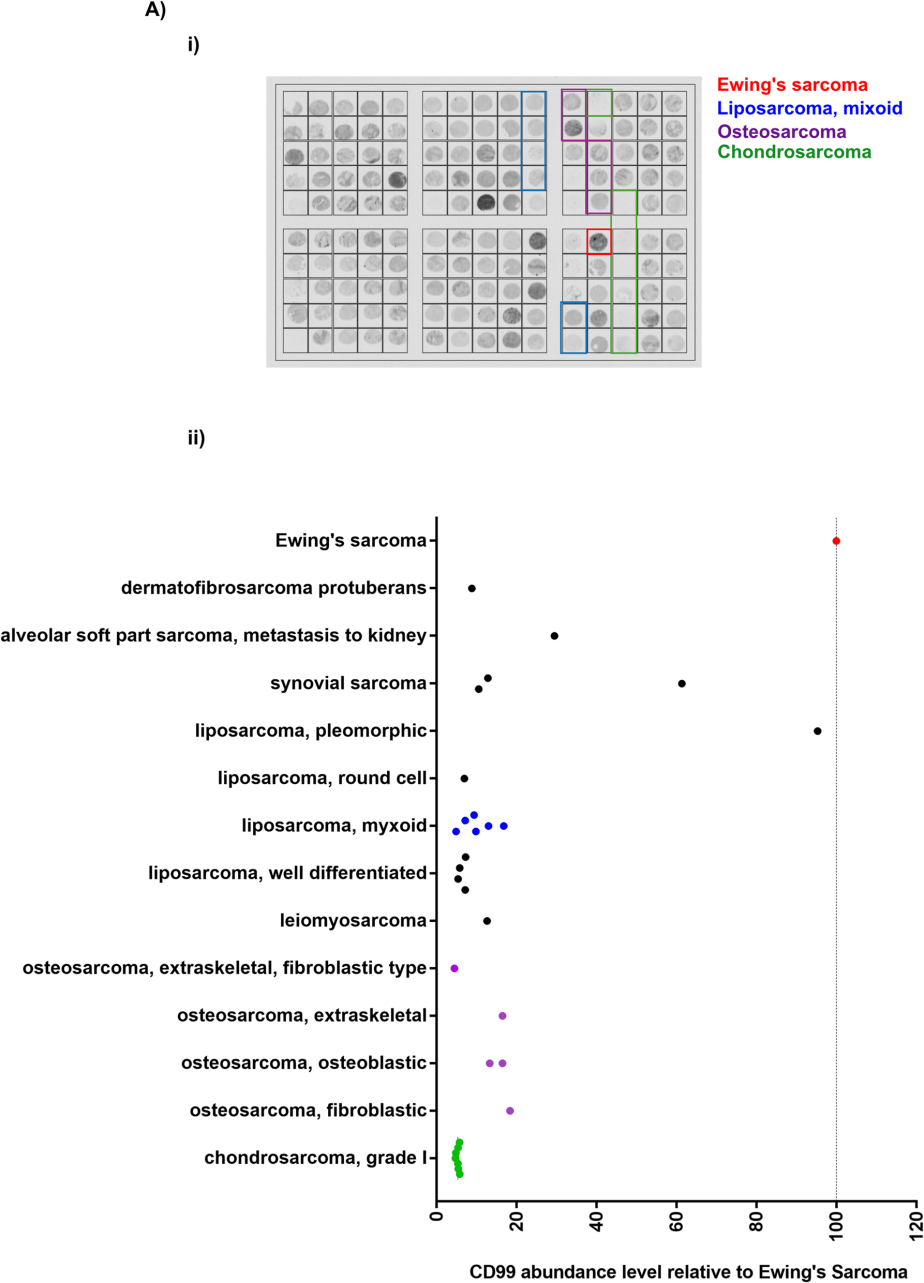
Myxoid liposarcoma tissues present low levels of CD99 expression.** A** Representative image of tissue microarray staining of CD99 in Ewing’s sarcoma, liposarcoma myxoid, osteosarcoma, and chondrosarcoma taken with Objective 10 ×. **B** Quantification of CD99 signal in cancer tissue microarray. Each dot represents one patient-derived sample, where the signal was normalized to Ewing’s sarcoma signal, and then regrouped by cancer type. *N* = 2 independent experiments were averaged. Ewing’s sarcoma is represented by a dotted line at 100% and serves as a positive control for CD99 abundance level as previously reported [20]

Using tissue microarrays combined with immunolabeling, we studied the level of expression of CD99 among a panel of sarcoma subtypes (Fig. 4). Ewing sarcoma tissue was used as a positive control in these assays since it has been previously reported to have a high level of CD99 immunoreactivity [20, 21]. Relative to Ewing sarcomas, CD99 immunostaining was noticeably decreased in most sarcoma subtypes including liposarcomas, osteosarcomas, and chondrosarcomas. In fact, only the pleomorphic liposarcoma tissue sample showed CD99 immunostaining comparable to that of Ewing sarcomas. Together, these results provide additional evidence of the potential relevance of decreased CD99 expression as a marker of sarcomagenesis.

## Discussion

MSCs constitute the stem cell population responsible for maintaining the homeostasis of mesenchymal tissues. It has been proposed that the deregulation of osteogenic, adipogenic, or chondrogenic differentiation processes due to stressful physiological or pathological situations may result in the appearance of premalignant MSCs/progenitor cells, which could eventually initiate sarcoma growth [1, 22, 23]. Following the initiation steps, tumors may start gaining complexity through the emergence of subpopulations of CSCs which usually share phenotypes and functional properties with MSCs, thus suggesting a likely MSC-origin of these subpopulations [1, 6]. Therefore, MSC transformation-based sarcomagenesis models represent unparalleled systems for unravelling the mechanisms underlying the sarcoma initiation process, exploring the evolution of CSC subpopulations and designing specific therapies capable of targeting the populations of the tumors able to re-initiate and sustain tumor growth.

The stepwise transformation model used in this work was generated by introducing oncogenic events that are known to play relevant roles in sarcomagenesis and can induce a gradual genomic hypomethylation of MSCs [24]. Thus, the mutation/deregulation of p53 and Rb signaling, the presence of genomic aberrations in TERT, the amplification of MYC, or the presence of activating mutations in different components of RAS signaling are common features of both soft tissue and bone sarcomas [25–27]. Proving the utility of this MSC-based system to model sarcomagenesis, we previously found that the expression of the fusion oncogene FUS-CHOP in these immortalized/transformed MSCs was able to trigger lineage instructive roles and cooperate with the transforming oncogenic hits to drive the formation of MLS in vivo [3]. Moreover, the introduction of other oncogenic drivers such as Mouse Double Minute (MDM) and Cyclin-dependent kinase 4 (CDK4) [28] or different components of the Activator Protein-1 complex [4] in this MSC-based model was able to respectively promote the growth of dedifferentiated liposarcomas or osteosarcomas. The work presented here further validates the relevance of this model through the direct comparison of protein abundance levels in sarcoma-forming cells from this model with tissue biopsies and samples from human sarcomas.

Proteins associated with MSC-directed sarcomagenesis could, therefore, be valuable in identifying molecular processes related to the transformation of MSCs and elaborating new treatment strategies against sarcomas. Through a mass spectrometry-based proteomics approach, we identified ALDH1A3 as a protein marker of interest and selected it for further validation based on previous research by our group showing that the expression and enzymatic activity of ALDH1 members correlated with increased CSC subpopulations and malignant potential in a tumor progression model of Myxoid Liposarcoma (MLS) [8]. We specifically validated ALDH1A3 increased abundance levels and activity from the immortalized and transformed MSC groups. Relevantly, ALDH1A3 has been described as a stemness marker, and its expression is associated with advanced disease in different types of solid tumors [29, 30]. This enzyme regulates several metabolic and detoxifying pathways that may favor the survival of both normal and CSCs, and its deregulated expression in cancer may result in excessive proliferation, chemoresistance, and invasiveness in colorectal cancer, breast cancer, or glioblastoma, among other types of cancer [31–34]. Indeed, the use of specific inhibitors of ALDH1A3 as a therapeutic strategy in cancer is being actively investigated [31, 32, 35]. The specific role of this ALDH1 isoform in sarcomas has been barely studied, and so far, only one report has involved ALDH1A3 in chemoresistance and metastatic dissemination in osteosarcoma [36]. Our findings suggest that the upregulation of ALDH1A3 may play relevant roles in the process of sarcoma initiation and the emergence of CSC subpopulations and, therefore, could represent a therapeutic target that deserves further research in sarcomas.

CD99 was also identified as a target of interest and was further validated to be downregulated in the MSC-transformation model, MLS-patient derived line in addition to human sarcoma subtype tissues. CD99 antigen, also known as Mic2, is a key surface glycoprotein used in T cell recognition and signalling, where co-ligation of CD99 and CD3 markedly enhances T cell activation [37]. Several studies indicate that CD99 is the primary adhesion molecule to generate an immunological synapse used by T lymphocytes to identify and regulate cell survival via cellular-mediated immune responses [38–40]. As reported previously by our group, the transformed MSCs developed a pro-inflammatory secretory profile associated with loss of immunosuppressive ability in support of tumor proliferation [5]. It can be suggested that the reduced abundance level of CD99 in the transformed cells could favor tumor progression by maintaining a low cellular infiltration despite their pro-inflammatory profile. Such a concept has been suggested for Hodgkin’s lymphoma Reed-Sternberg cells (HLRSCs), where the downregulation of CD99 expression provides more resistance to cytotoxic T lymphocytes (CTLs) and apoptosis; thereby favoring the HRSCs’ survival [41]. In this line, genetic analysis of the transformed BM-hMSCs previously identified the increased expression of immune checkpoint molecules such as Programmed Death-Ligand 1 (CD274/PD-L1) and CD86 which could play a role in inhibiting the activation of infiltrating T cells [5]. Binding partners of CD99 include self-dimerization with adjacent cells [42], co-localization with integrin proteins and other immune regulatory antigens such as CD81 and Major Histocompatibility Complex (MHC) class receptors to form the immunological synapse [40]. It could be suggested that reduced surface abundance of CD99 may be associated with poor immune recognition. For example, MLS patient tumor samples observed some of the lowest concentrations of tumor infiltrating lymphocytes [43], supporting the potential lack of leukocyte cellular adhesion [44]. Validation of reduced CD99 abundance patterns in patient tumor-derived MLS cells, DL-221, originally characterized by de Graaff et al. [45], aids in supporting its connection to sarcomagenesis.

## Conclusions

This study provides insights into CD99 and ALDH1A3’s role as potential markers of MSC transformation and sarcomagenesis. Overall, this work could broaden our understanding of the disease, which may contribute to the development of novel therapeutic approaches for sarcomagenesis.

## Methods

### Cell culture

The wild type BM-hMSC culture hBM#44 was obtained from Inbiobank (San Sebastian, Spain) and were functionally and phenotypically characterized in a previous work [5]. hBM#44-GFP were obtained after transducing hBM#44 cells with lentiviral particles carrying the GFP-expressing vector pRRL-EF1a-FUS-CHOP-PGK-GFP [3]. hBM12A and hBM15A cultures were derived from mononuclear cells (cat.#1 M-125D) isolated by Lonza (Lonza, Walkersville, MD, USA) with informed consent, and all BM-hMSC cultures were grown in 15% MSC-screened FBS (Corning, Glendale, AZ; cat.#35–079-CV) in DMEM (Corning; cat.#10–017-CV) [46]. hBM#7043, hBM#7083 and hBM#8004 refer to cultures obtained from the Texas A&M Health Science Center College of Medicine Institute for Regenerative Medicine at Scott & White previously reported here [47]. The BM-hMSCs carrying the different oncogenic hits (MSC-3H, MSC-4H, and MSC-5H) were developed and characterized elsewhere [2]. FUS-CHOP was overexpressed in these cell lines by transducing them with lentiviral particles expressing either the pRRL-EF1a-PGK-GFP vector (empty vector) to obtain the MSC-3H-GFP, MSC-4H-GFP, and MSC-5H-GFP cells or the pRRL-EF1a-FUS-CHOP-PGK-GFP vector (FUS-CHOP (FC) expressing vector) to generate MSC-3H-FC, MSC-4H-FC, and MSC-5H-FC cells, as previously reported [3]. The human myxoid liposarcoma cell line (DL221) was obtained from MD Anderson Cancer (cat.#42) and characterized elsewhere [45]. Immortalized and transformed MSCs were cultured in DMEM (Gibco, cat.#10,566–016) supplemented with 15% MSC-screened FBS HyClone (Gibco, cat.#SH30070.M). The DL221 cells were cultured in DMEM (Gibco, cat.#10,569–010) supplemented with 15% FBS (Gibco, cat.#12,483–020). All lines were placed in a humidified 5% CO_2_ incubator at 37 °C.

### BM-hMSC protein lysate preparation for TMT labeling for mass-spectrometry proteomics analysis

Lysis buffer (100 mM TEAB with 1% SDS) was prepared according to the manufacturer’s protocol using the reagents provided in the TMT labeling kit (TMT 10plex Mass Tag Labeling kits and Reagents Thermo, cat.#90,113). The cells were incubated with lysis buffer for proper lysing for 30 min at 4 °C on an end-over-end shaker (LabQuake Shaker) and then centrifuged down for 30 s at 1000 rpm after incubation. The protein lysates were sonicated (Fisher Scientific, Model#FB120) at amplitude setting 30% for 3 × 10 s with 30 s on ice between pulses. After sonication, samples were centrifuged at 14,000 × g for 5 min at 4 °C. The supernatant was recovered and stored in 1.5 mL Protein LoBind tubes at − 80 °C. Prior to freezing at − 80 °C, an aliquot was taken out for protein quantification by the bicinchoninic acid (BCA) assay (Pierce BCA Protein assay kit, cat.#23,227). Samples were kept frozen at − 80 °C for downstream analysis. The TMT protocol was followed according to the manufacturer’s protocol, with slight modifications. Refer to the TMT 10plex Mass Tag Labeling Kits and Reagents (Thermo Scientific, cat.#90,113) for the product description and to our previous work [47].

### LC–MS-MS analysis

All peptide MS spectra were acquired on a Thermo Scientific EASY-nLC 1000 system coupled to a Thermo Scientific Orbitrap Fusion Lumos Tribrid mass spectrometer using a 25-cm × 75-µm ID EASY-Spray 2-µm C18 fitted column into EASY-Spray Ion Source. Mobile phase A used was 0.1% formic acid in water, while mobile phase B was 0.1% formic acid in acetonitrile. The separation of peptides was done by increasing mobile phase B from 5 to 25% over 112 min period followed by 25–60% for 20 min and finally 60–90% over 5 min. For peptide identification and quantitation, SPS MS3 was performed using a FTMS full scan (resolution 120,000 @ m/z 200, scan range of 400–1500 m/z, maximum injection time of 50 ms, AGC target 4E5, microscan of 1 and RF set to 30%) followed by IT MS2 CID (Isolation was performed in the quadrupole with a window of 0.7 using collision energy of 35%. Maximum injection time of 50 ms was used along with AGC target of 1E4) and FT MS3 HCD (resolution 30,000 @ m/z 200, using isolation of 0.7 at collision energy 65%. Mass range was set to 100–500 m/z using maximum injection time of 105 ms with AGC target of 1E5) on a total of 10 fragments from the MS2 spectra. Data analysis was performed using Proteome Discoverer v2.0 (Thermo Fisher Scientific) and was used for peptide searches. The raw files were searched against a Uniprot_sprot.FASTA database (parsed for human taxonomy) using the SEQUEST search algorithm. Proteins were identified based on a false discovery rate (FDR) of < 1% using the Percolator validator node. Peptide searches allowing up to two missed cleavages were performed using 10-ppm precursor tolerance and 0.6-Da MS/MS tolerance. Cysteine carbamidomethylation and TMT 10-plex of lysine and peptide N termini were set as static modifications. Asparagine and glutamine deamidation and methionine oxidation were set as dynamic modifications. Peptide quantitation was performed at the MS3 level. Peak integration tolerance was 10 ppm with the highest confidence centroid selected. Reporter ion intensities were adjusted to correct for the isotopic impurities of each TMT reagent (according to the manufacturer’s specifications). Mass spectrometry data is reported in Additional file 9: Table S6. The mass spectrometry proteomics data have been deposited to the ProteomeXchange Consortium via the PRIDE partner repository [48] with the dataset identifier PXD069892 and DOI 10.6019/PXD069892 [49, 50].

### Statistical analysis of the mass spectrometry proteomic datasets and pathway enrichment analysis

The differential expression analyses were applied using the *t*-test; a Satterthwaite approximation was adopted whenever unequal variance was evident. The fold change is defined as the ratio of means (geometric mean for log2 transformed data). The differentially expressed proteins were identified using the raw *p*-value (alpha level of < 0.05) and a fold change greater than 1.50 (± 1.50). The raw *p*-values were used for generating the volcano plots, and the FDR adjusted *p*-values are also provided in the supplementary document for reference (Additional files 3–5: Tables S3-S5). The proteins with 100% missing values in comparisons (I vs N, T vs N, T vs I) were excluded from the analyses. Initially, 3099 proteins were identified, and after the exclusion, there were 2585 proteins for the Immortalized vs Normal and the Transformed vs Immortalized comparisons, and 2584 proteins for the Transformed vs Normal comparison. Missing values were imputed using the minimal value of the corresponding protein. The analyses were implemented using SAS Enterprise Guide 5.1 [51]. The protein datasets were further analyzed by the Ingenuity Pathway Analysis (IPA) curated database for biological significance using the pathway enrichment tools for downstream analysis. Diseases and biological functions exhibiting high activation *z*-scores involved proteins of interest, ALDH1A3 and CD99, were prioritized for pathway mapping. IPA’s core analysis was used to identify direct and indirect interactions between these proteins and associated biological processes. Predicted activation or inhibition states were inferred based on the expression values in the dataset, using IPA’s curated molecular interaction database and upstream regulator analysis.

### Western blot analysis

Forty micrograms of whole cell lysate were combined with 4 × LI-COR Protein Loading Buffer (Li-Cor, cat.#928–40,004), Bolt™ 10 × Reducing Agent (ThermoFisher Scientific, cat.#B0009), denatured, and run in Bolt™ Bis–Tris 4%–12% 17 well SDS-PAGE (ThermoFisher Scientific, cat.#NW04127BOX). Gels were transferred to Immobilon™ FL PVDF membranes (ThermoFisher Scientific, cat.#88,025) using Bolt™ Mini Module, stained with LI-COR REVERT total protein stain (cat.#926–11,010), and imaged using LI-COR Odyssey CLx (Auto intensities 700/800). Blots were incubated with antibodies using the iBind Flex Western system (ThermoFisher Scientific, cat.#SLF1000) and re-imaged for band intensities. Antibodies were obtained from Novus Biologicals (ALDH1A3, cat.#NBP2-46,510), (CD99, cat.#NBP2-46,181), Genetex (GAPDH, cat.#GTX627408), and LI-COR Biosciences (IRDye™ 800CW, cat.#925–32,210 and IRDye™ 680RD, cat.#926–68,071). Fluorescent band intensity was normalized using total lane signal observed from REVERT™ Total Protein Stain. Relative quantification was done by normalizing the highest intensity of the 800 nm channel to 100% abundance.

### ALDEFLUOR assay

ALDH activity was determined using the activated Aldefluor™ reagent (Stem Cells Technologies, Grenoble, France, cat.#01700) as previously reported [6, 13]. Briefly, 1 × 10^6^ cells were suspended in 1 mL of Aldefluor assay buffer containing the ALDH1 substrate (Bodipy-Aminoacetaldehyde) and incubated for 45 min at 37 °C. As a gating control, cells were suspended in buffer containing the substrate in the presence of the specific ALDH1 enzyme inhibitor diethylaminobenzaldehyde (DEAB; 10 µM). Cells were incubated with 0.5 µg/mL propidium iodide for 15 min, and cells positive for this staining (dead cells) were excluded from the analysis. Gates were established to include less than 0.3% positive cells in the DEAB controls.

### Immunostaining of cell surface CD99

Cells were seeded at 15,000 cells/well in 96-well plates in DMEM with 15% FBS and let to adhere overnight before being fixed using 4% PFA. The cells were blocked and incubated with anti-human CD99 (Novus Biologicals, cat.#NBP2-46,181) primary antibody overnight at 4 °C. IRDye 800CW secondary antibody (Li-Cor Biosciences, cat.#925–32,210) was incubated for 4 h at room temperature. Plates were imaged using an Infrared Imaging System Odyssey CLx from Li-Cor. Afterwards, cells were permeabilized with 0.1% Triton X-100 and stained with 0.2 µM CellTag 700 (Li-Cor Biosciences, cat. 926–41,090) for normalization. Relative quantification was done by normalizing the highest intensity of the 800 nm channel to 100% abundance.

### Immunostaining of tissue microarray

The tissue microarray (#NBP2-30,330) was purchased from Novus Biologicals (Toronto, ON, Canada). Respective and detailed clinical data of the tissue samples used can be found at the following vendors’ website: https://www.novusbio.com/products/various-tissue-micro-array_nbp2-30330; web site accessed on 24 August 2022). Tissue microarray slides were heated to 60 °C for 1 h. After deparaffinization with 5 changes in xylene for 4 min each, the sections were hydrated in graded ethanol solutions to distilled water. The slides were washed twice in PBS for 5 min and then incubated with blocking buffer (Li-Cor Biosciences, cat.#927–40,003) for 2 h at room temperature, followed by incubation with the primary antibody (Novus Biologicals, cat.#NBP2-46,181) overnight at 4 °C. After washing with the blocking buffer, the secondary antibody (Li-Cor Biosciences, IRDye 800CW goat anti-mouse IgG secondary antibody, cat.#926–32,210) was diluted in blocking buffer (1:4000) and incubated for 1 h at room temperature. The slides were rinsed in water for 5 min before being imaged on the Li-Cor Odyssey CLx (21 µm, highest resolution, 1.0 mm offset).

### Statistics

Statistical significance was analyzed by a one-way analysis of variance (ANOVA) with Tukey’s post hoc analysis as indicated in the figure legends. Statistical analyses were performed using GraphPad Prism version 6.0 (GraphPad Software Inc., La Jolla, CA, USA). A *p*-value of < 0.05 was considered statistically significant and significance differences are marked with a single (*p* < 0.05), double (*p* < 0.01), or a triplicate (*p* < 0.001) asterisk.

## Supplementary Information

Additional file 1.

Additional file 2.

Additional file 3.

Additional file 4.

Additional file 5.

Additional file 6. Figure S1. Full-length Western blot of ALDH1A3, CD99 and GAPDH. A) A representative full-length Western blot of ALDH1A3, CD99 and GAPDH is shown using MSCs from immortalized (n = 3) and transformed (n = 3) MSC samples.

Additional file 7. Figure S2. Full-length plate immunoassay scanning of CD99 and Total Protein Stain (TPS) signals. Representative images of 4 plates scanned for immunostaining of cell surface CD99 signal (infrared IRDye 800 channel; green color) and total protein stain (TPS) (infrared Cell Tag 700; red color) with cell lines seeded as indicated (N = normal, I = immortalized, T = transformed, MLS = myxoid liposarcoma).

Additional file 8. Figure S3. CD99 and ALDH1 transcriptomic expression in MSC cells. A) Violin plot of ALDH1A3 and CD99 genes showing single-cell expression distribution in MSCs from healthy adults (SAA; n = 3) and healthy infants (SAI; n = 4). B) Dot plot of ALDH1A3 and CD99 genes showing single-cell expression in MSCs from healthy adults (SAA) and healthy infants (SAI). The size of circle represents the proportion of single cells expressing the gene, and the color shade indicates normalized expression level.

Additional file 9.

## Data Availability

The datasets generated and/or analyzed during the current study are available in the Supplementary section. The mass spectrometry proteomics data have been deposited to the ProteomeXchange Consortium via the PRIDE partner repository [48] with the dataset identifier PXD069892 and DOI 10.6019/PXD069892 [49, 50].

## Abbreviations

ALDH: Aldehyde dehydrogenase
ALDH1A1: Aldehyde dehydrogenase 1 family member A1
ALDH1A3: Aldehyde dehydrogenase 1 family member A3
BM: Bone marrow
BM-hMSCs: Bone marrow-derived human mesenchymal stromal/stem cells
CDK4: Cyclin-dependent kinase 4
CSC: Cancer stem cells
CTLs: Cytotoxic T lymphocytes
EGF: Epidermal growth factor
EIF2: Eukaryotic Initiation Factor 2
FDR: False discovery rate
HLRSCs: Hodgkin’s lymphoma Reed Sternberg cells
hBM: Human bone marrow
I: Immortalized
IL-8: Interleukin-8
ILK: Integrin-linked kinase
IPA: Ingenuity pathway analysis
LC–MS-MS: Liquid chromatography with tandem mass spectrometry
MDM: Mouse double minute
MHC: Major histocompatibility complex
MLS: Myxoid liposarcomas
MS: Mass spectrometry
MSC: Mesenchymal stromal/stem cells
N: Normal
PD-L1: Programmed death-ligand 1
Rb: Retinoblastoma
T: Transformed
TERT: Telomerase reverse transcriptase
TMT: Tandem Mass Tag
scRNAseq: Single cell RNA sequencing
