# Supplementary material for: Proteomics analysis of human mesenchymal stromal/stem cell sarcomagenesis model identifies ALDH1A3 and CD99 as potential targets in the transformation process
Source: BMC Biol. 2026 Jan 9;24:32. doi: 10.1186/s12915-025-02498-z (PMC12882515; doi:10.1186/s12915-025-02498-z)
Supplement: Supplementary file 1 — Additional file 1. [file 12915_2025_2498_MOESM1_ESM.docx]

| **Table S1. Summary of the oncogenic status and transforming mutations of the BM-hMSC cells.** | | | | | |  |
| --- | --- | --- | --- | --- | --- | --- |
| **hBMSC line** | **Included in proteomic analysis (Abbreviation)** | **Transforming hits** | **FUS-CHOP expression** | **GFP expression** | **Oncogenic status** | **Sarcoma model** |
| **hBM12A** | Yes (N1) | none | No | No | wild type | - |
| **hBM15A** | Yes (N2) | none | No | No | wild type | - |
| **hBM#44** | Yes (N3) | none | No | No | wild type | - |
| **hBM#44-GFP** | Yes (N4) | none | No | Yes | wild type | - |
| **hBM#7043** | No (N5) | none | No | No | wild type | - |
| **hBM#7083** | No (N6) | none | No | No | wild type | - |
| **hBM#8004** | No (N7) | none | No | No | wild type | - |
| **MSC-3H** | No | p53 deficiency / Rb deficiency / hTERT overexpression | No | No | immortalized | - |
| **MSC-3H-GFP** | Yes (I1) | p53 deficiency / Rb deficiency / hTERT overexpression | No | Yes | immortalized | - |
| **MSC-3H-FC** | Yes (I2) | p53 deficiency / Rb deficiency / hTERT overexpression | Yes | Yes | immortalized | - |
| **MSC-4H-GFP** | Yes (I3) | p53 deficiency / Rb deficiency / hTERT overexpression / c-myc stabilization | No | Yes | immortalized | - |
| **MSC-4H-FC** | Yes (T1) | p53 deficiency / Rb deficiency / hTERT overexpression / c-myc stabilization | Yes | Yes | transformed | Myxoid liposarcoma |
| **MSC-5H** | No | p53 deficiency / Rb deficiency / hTERT overexpression / c-myc stabilization / RAS^v12^ | No | No | transformed | Undif. spindle cell sarcoma |
| **MSC-5H-GFP** | Yes (T2) | p53 deficiency / Rb deficiency / hTERT overexpression / c-myc stabilization / RAS^v12^ | No | Yes | transformed | Undif. spindle cell sarcoma |
| **MSC-5H-FC** | Yes (T3) | p53 deficiency / Rb deficiency / hTERT overexpression / c-myc stabilization / RAS^v12^ | Yes | Yes | transformed | Myxoid liposarcoma |
| **DL221** | No | Patient-derived | Yes | No | transformed | Myxoid liposarcoma |
