# Supplementary figures and images for: Proteomics analysis of human mesenchymal stromal/stem cell sarcomagenesis model identifies ALDH1A3 and CD99 as potential targets in the transformation process
Source: BMC Biol. 2026 Jan 9;24:32. doi: 10.1186/s12915-025-02498-z (PMC12882515; doi:10.1186/s12915-025-02498-z)

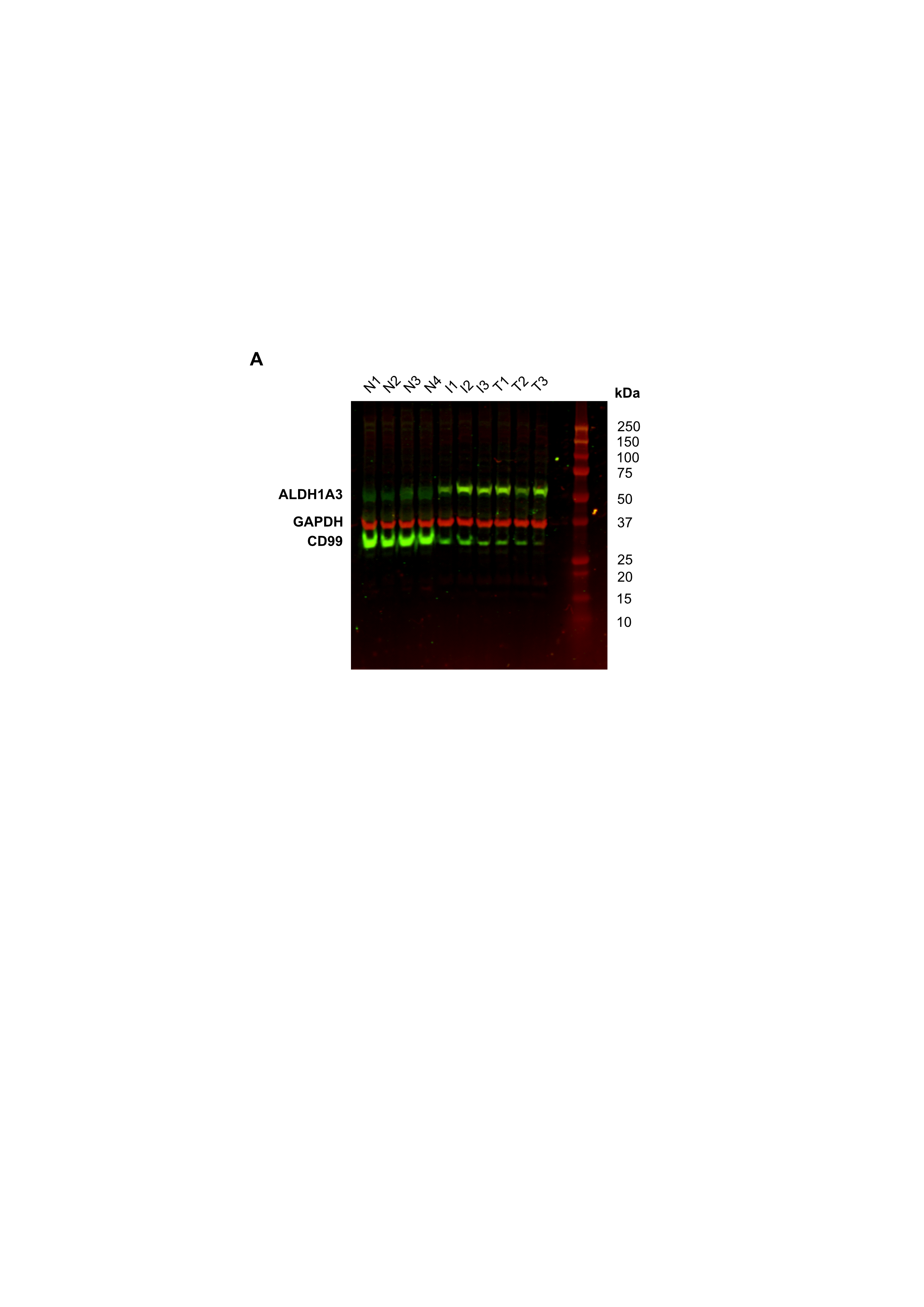

Supplement: Supplementary file 6 — Additional file 6. Figure S1. Full-length Western blot of ALDH1A3, CD99 and GAPDH. A) A representative full-length Western blot of ALDH1A3, CD99 and GAPDH is shown using MSCs from immortalized (n = 3) and transformed (n = 3) MSC samples. [file 12915_2025_2498_MOESM6_ESM.tiff]

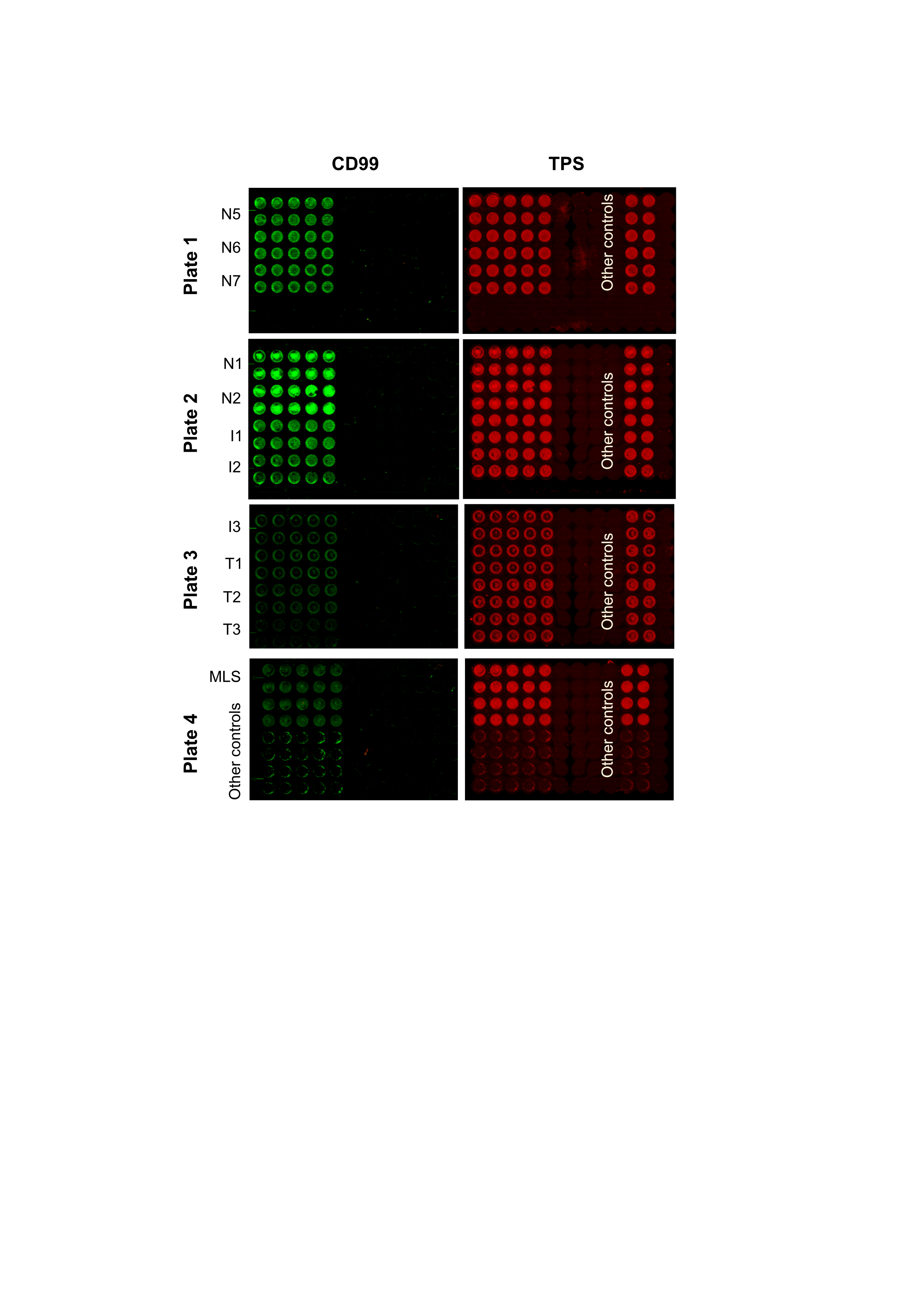

Supplement: Supplementary file 7 — Additional file 7. Figure S2. Full-length plate immunoassay scanning of CD99 and Total Protein Stain (TPS) signals. Representative images of 4 plates scanned for immunostaining of cell surface CD99 signal (infrared IRDye 800 channel; green color) and total protein stain (TPS) (infrared Cell Tag 700; red color) with cell lines seeded as indicated (N = normal, I = immortalized, T = transformed, MLS = myxoid liposarcoma). [file 12915_2025_2498_MOESM7_ESM.tiff]

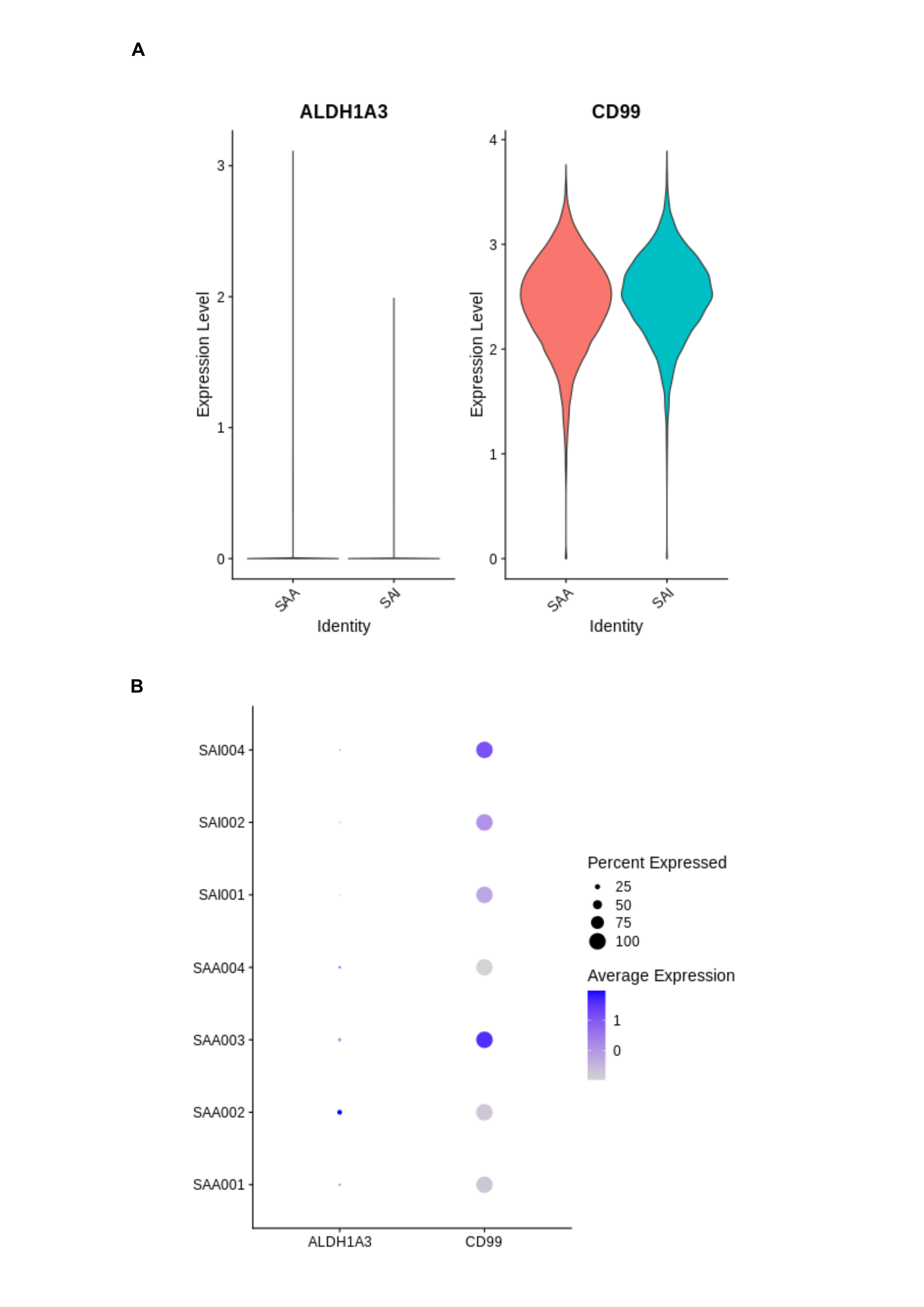

Supplement: Supplementary file 8 — Additional file 8. Figure S3. CD99 and ALDH1 transcriptomic expression in MSC cells. A) Violin plot of ALDH1A3 and CD99 genes showing single-cell expression distribution in MSCs from healthy adults (SAA; n = 3) and healthy infants (SAI; n = 4). B) Dot plot of ALDH1A3 and CD99 genes showing single-cell expression in MSCs from healthy adults (SAA) and healthy infants (SAI). The size of circle represents the proportion of single cells expressing the gene, and the color shade indicates normalized expression level. [file 12915_2025_2498_MOESM8_ESM.tiff]
